# Sagnac Effect Compensations and Locked States in a Ring Laser Gyroscope

**DOI:** 10.3390/s23031718

**Published:** 2023-02-03

**Authors:** Woo-Seok Choi, Kyu-Min Shim, Kyung-Ho Chong, Jun-Eon An, Cheon-Joong Kim, Byung-Yoon Park

**Affiliations:** 1 Agency for Defense Development, Daejeon 34186, Republic of Korea; 2 Department of Physics, Chungnam National University, Daejeon 34134, Republic of Korea

**Keywords:** Sagnac interferometry, ring laser gyroscope, frequency lock-in, deadband, Sagnac effect compensation

## Abstract

Frequency lock-in-induced deadband phenomena are major problems of ring laser gyroscopes (RLGs), which deteriorate linear responses to changes in the applied rotation rate. In this work, the frequency lock-in phenomenon occurring in the RLG was successfully investigated by compensating for the Sagnac effect through frequency analysis using a newly defined error function. Integrative and generalized viewpoints from the analyzed results provide new possibilities for relevant performance improvements of optical gyroscopes, as well as a deeper understanding of locked states in principle aspects.

## 1. Introduction

The ring laser gyroscope (RLG) is a representative angular velocity sensor [1] along with the other modern gyro technologies based on such as the optical fiber interferometer [2], the semiconductor waveguide ring resonator [3] or the microelectromechanical systems (MEMS) [4]. It is widely used in various fields, such as inertial systems that require precision performance at the navigation level. To guarantee the precise performance of an RLG, it is necessary to identify performance-related error factors and find a way to solve them. When a rotational physical quantity below a certain threshold is applied to the RLG, the frequency difference between the light beams traveling in the clockwise (CW) and counterclockwise (CCW) directions is negligible, known as the frequency lock-in phenomenon of the RLG. It is the most important error factor affecting performance, and finding a solution has been a major part of the efforts toward RLG development. To minimize the error factor due to frequency lock-in, a dithering technique that mechanically applies an alternating rotational physical quantity to the ring laser gyro body has been proposed and applied extensively and successfully [5,6,7,8,9,10,11]. However, based on the results of previous studies, when the frequency lock-in width is relatively large, or there is a restriction when applying a fast and strong dithering force, it remains a challenge to secure precise performance despite applying the dithering technique. Therefore, it is essential to find a new method based on a deeper understanding of the frequency lock-in phenomenon to improve the performance of the RLG.

In this study, a new error function was defined through the lock-in equation to explain the frequency lock-in phenomenon as well as the mechanism of the error function compensating for the Sagnac effect occurring in proportion to the rotational input angular velocity at the RLG output level. The results of numerical experiments to confirm this relationship were presented. This analysis method enabled a deeper understanding of the field of frequency lock-in and relevant nonlinear phenomena at a fundamental level; it can simultaneously contribute to finding new methods for improving the performance of the RLG in a more generalized category.

## 2. Sagnac Effect for the RLG

The operating principle of an optical gyro, such as a ring laser gyro or fiber-optic gyro, is based on the Sagnac effect [8,12,13,14]. Two light beams traveling in the CW and CCW directions along a circular optical path from the starting point meet exactly at the starting point in a stationary state where rotation is not applied. By contrast, when rotation is applied to an object, including a circular optical path, as expressed in Equation (1), a difference in the round-trip optical path proportional to the applied rotational physical quantity occurs. Unlike in the stationary state, the light beams meet at a point other than the starting point.

(1) $$\Delta l=\frac{4\pi \rho^{2}\Omega }{c}\;,$$

where Δl is the round-trip optical path difference; ρ is the radius of the circular beam path; Ω is the angular velocity; and ***c*** is the speed of light. If the Sagnac effect is generalized to a structure in the form of an arbitrary closed optical path rather than an ideal circular optical path, it can be verified that the round-trip optical path difference owing to the rotational physical quantity increases in proportion to the area enclosed by the closed path, as expressed in Equation (2).

(2) $$\Delta l=\frac{4\Omega \cdot \hat{Z}A}{c}\;,$$

where A is the area enclosed by the light path and $\hat{Z}$ is a unit vector normal to the surface of the interferometer. Particularly, in the case of an RLG having the form of an active resonator, a difference in the effective resonator length occurs because of the rotation applied between the beams propagating in the CW and CCW directions. Therefore, when the laser resonance condition is maintained in the RLG, an angular frequency difference a occurs between the two beams traveling in opposite directions owing to the Sagnac effect, as expressed in Equation (3).

(3) $$a=\frac{8\pi A\Omega }{L\lambda }\;,$$

where ***L*** is the perimeter of the light path and ***λ*** is the wavelength of the laser light.

## 3. Frequency Lock-In Dynamics in the RLG

The main cause of the frequency lock-in phenomenon is backscattering from the mirrors that constitute the ring laser. If there is a backscattering component in the laser resonator, the frequency difference between the beam traveling in the CW direction and the beam traveling in the CCW direction in the ring laser optical path cannot be expressed simplistically by Equation (3), and a theoretical analysis considering the cross-coupling characteristics is required. At present, the mathematical theory explaining the frequency lock-in phenomenon of the RLG is well-established, and the results have been successfully verified through numerical calculations and actual experiments [5,6,7,8,9,10,11]. In this section, with reference to the same notation as in [8], a summary and arrangement of the related content will be presented. The RLG frequency lock-in equation, showing the effect of backscattering, is well-established and can be expressed as Equation (4).

(4) $$\dot{\psi }=a+bsin\psi \;,$$

where ψ is the phase difference with a unit of radian between the CW and CCW propagating laser beams and b is the backscattering coefficient, which is expressed as

(5) $$b=\frac{\pi \nu \chi_{0}r_{s}}{\varepsilon_{0}}\;,$$

where ***ν*** is the optical frequency that is obtained with the dispersive effects of the active medium; $\chi_{0}$ and $\varepsilon_{0}$ are the dielectric susceptibility and vacuum permittivity, respectively; and $r_{s}$ is the amplitude of the coupling factor owing to backscattering. Equation (4) can be analytically divided into two parts and examined according to the relationship between a and b when determining the value of ψ. That is, when a is smaller than ***b***, the right side of Equation (4) becomes zero, regardless of the value of a, even though a is not zero. In this case, the frequencies between the two beams traveling in opposite directions inside the ring laser resonator are locked to the same value. By contrast, when a is larger than b, the right side of Equation (4) is outside the frequency-lock state and has a nonzero value corresponding to a. If the average period ***T*** for $\dot{\psi }$ is defined, it can be expressed as Equation (6).

(6) $$T\equiv \int_{0}^{2\pi }\frac{d\psi }{a+bsin\psi }=\frac{2\pi }{\sqrt{a^{2}-b^{2}}}\;$$

As shown in Equation (7), the average angular frequency difference $\Delta \omega_{out}$ in the unlocked region recognized by the RLG can be defined in response to a.

(7) $$\Delta \omega_{out}\equiv \Gamma =\sqrt{a^{2}-b^{2}}\;$$

From Equation (7), when the difference between the a and b values is not large, the change in the average frequency difference with respect to ***a*** shows a nonlinear relationship characteristic owing to the square root function relationship. However, when the difference between the a and b values is sufficiently large, the average angular frequency difference converges to a, showing a linear relationship.

Therefore, if the value of b is large, the frequency lock-in region is widened, making it difficult to implement a precise RLG in the low rotational input angular velocity region. As noted in previous research, values of b as large as 1000 rad/s are common in regard to real-life development and application [8], whereas the error level of the ring laser gyro according to the b value is far from the level required by the navigation grade system. For this reason, finding a way to reduce the effect of frequency lock-in, even in a situation with a relatively high b value, has been prioritized in the development history of the RLG. The dithering technique, which mechanically applies periodic angular vibration in the form of a sinusoidal wave to the body of an RLG, is the most widely and successfully applied method to solve the problem caused by frequency lock-in. This is usually done by the angular piezoelectric deformation to oscillate the gyro body about the rotation axis [15]. When sinusoidal dithering is included, the previous frequency lock-in equation can be rewritten as Equation (8).

(8) $$\dot{\psi }=a+bsin\psi +\alpha cos\left(\omega_{D}t\right)\;,$$

where α and $\omega_{D}$ are the amplitude and angular frequency of the sinusoidal dithering waveform, respectively. In principle, changing the cosine function for the dithering term in Equation (8) to a sine function is equivalent to the time translation of $t\to t+\pi /\left(2\omega_{D}\right)$, and the phase difference increments should not depend on this change. However, if the sine function is used instead of the cosine function for the dithering term, it is inevitable that ψ in Equation (8) has changes in the initial values which can cause slightly different features in the temporal evolution of ψ**,** according to the possible practical situations. The solution of Equation (8), ψ**,** can be expressed in the form of Equation (9).

(9) $$\psi \left(t\right)=\psi_{i}\left(t\right)+\delta \left(t\right)=at+\frac{\alpha }{\omega_{D}}sin\left(\omega_{D}t\right)+\delta \left(t\right)\;,$$

where δ is the solution of Equation (10).

(10) $$\dot{\delta }\left(t\right)=\overset{\infty }{\underset{m=-\infty }{\sum }}bJ_{m}\left(\frac{\alpha }{\omega_{D}}\right)sin\left[\left(a+m\omega_{D}\right)t+\delta \left(t\right)\right]\;,$$

where ***J_m_*** is the ***m***th Bessel function of the first kind. The sum of Equation (10) has a special meaning when a is near an integer multiple of $\omega_{D}$, as expressed in Equation (11).

(11) $$a=r\omega_{D}+\tilde{a}\;,$$

where $\left|\tilde{a}\right|\ll \omega_{D}$. That is, when m=−r, $\left(a+m\omega_{D}\right)=\tilde{a}$ and the related term changes slowly. However, when m≠−r, all other terms change rapidly to at least $\omega_{D}$ or more. Therefore, if the average is taken over several dithering cycles, only the term corresponding to m=−r remains; thus, by substituting $\varphi =\tilde{a}t+\delta \left(t\right)$, Equation (10) can be rewritten as:

(12) $$\dot{\varphi }=\tilde{a}+bJ_{-r}\left(\frac{\alpha }{\omega_{D}}\right)sin\left(\varphi \right)\;.$$

Therefore, by comparing Equations (4), (8) and (12), whenever a becomes an integer multiple of $\omega_{D}$ through dithering, a frequency lock-in region in which ψ does not change, despite changes in the value of a, is generated near that value. Meanwhile, the size of the lock-in region is reduced by $J_{-r}\left(\alpha /\omega_{D}\right)$ times compared to the original b.

**Figure 1:**
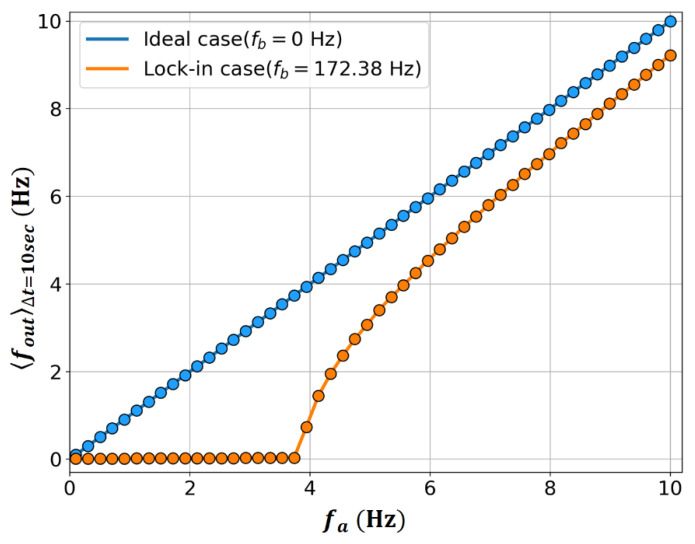
Numerically calculated RLG outputs $\langle f_{out}\rangle {}_{\Delta t}$ with sinusoidal dithering as a function of $f_{a}=a/2\pi$.

For the case of r=0, the related characteristic change due to dithering can be confirmed through a numerical experiment using Equation (8), and the results are shown in Figure 1. The parameters used in the numerical experiment were $b/2\pi =f_{b}=$172.38 Hz, $\alpha /2\pi =f_{\alpha }=$137.905 kHz, and $\omega_{D}/2\pi \;$= 800 Hz. The Runge–Kutta method was used to solve Equation (8). ${\langle \dot{\psi }\rangle }_{\Delta t}$, which is the average $\dot{\psi }$ during the observation time Δt**,** according to the change in a value, can be calculated using Equation (13) [16], and the RLG output $\langle f_{out}\rangle {}_{\Delta t}\;$in Figure 1 corresponds to the calculated result for Δt = 10 s,

(13) $$\langle f_{out}\rangle {}_{\Delta t}=\frac{{\dot{\psi }}_{\Delta t}}{2\pi }=\frac{\psi \left(t+\Delta t\right)-\psi \left(t\right)}{2\pi \Delta t}\;.$$

The used observation time of 10 s has 8000 dithering cycles, considering the dithering frequency, $\omega_{D}/2\pi$ is 800 Hz in this study. This large number of dithering cycles can ensure a high precision in the extraction of RLG outputs with the evolutional information on the phase difference, ψ in Equation (13). From Figure 1, typical frequency lock-in characteristics are confirmed when b≠0, unlike the ideal case at b=0. Additionally, the size of the frequency lock-in region that appears in this case is reduced to$\;f_{b}J_{0}\left(\alpha /\omega_{D}\right)\approx$ 3.87 Hz, as in Equation (12), instead of $f_{b}$ = 172.38 Hz.

## 4. Interpretation of Lock-In Mechanism with Sagnac Effect Compensation

How can the typical frequency lock-in phenomenon of the RLG shown in Figure 1 be interpreted in relation to the Sagnac effect discussed in Section 2? As a first step in explaining that the output of the RLG becomes zero even though a nonzero a clearly exists in the region where a is smaller than b, the following two different perspectives can be considered depending on whether a reinterpretation of the Sagnac effect is necessary.

First, there may be an interpretive view that the RLG frequency lock-in phenomenon is caused by the Sagnac effect itself being affected by the existence of a nonzero b; Equation (3) is the result obtained assuming that there are only two beams traveling in the CW and CCW directions inside the laser cavity, and the effect of cross-coupling between the two beams from backscattering is not considered. The new development of the Sagnac effect theory, including the effect of cross-coupling caused by the backscattering, can stimulate a compelling and new perspective in the field of physics itself, as well as explore various possibilities in terms of improving the performance of the RLG. However, a challenge remains in that a major revision of the well-established theory from the basic principle level is inevitable for this purpose.

In addition, there may be a phenomenological analysis in which the frequency difference due to the Sagnac effect in Equation (3) is compensated for by an error component generated by the existence of a nonzero b. The typical RLG frequency lock-in phenomenon shown in Figure 1 makes this interpretation intuitive. In this case, the RLG output with the frequency lock-in characteristic of the solid orange line adopts the viewpoint that the ideal RLG output of the solid blue line is compensated for by the corresponding error component. Alternatively, it may be interpreted as the gain in terms of the frequency difference; that is, the energy difference caused by the Sagnac effect is attenuated owing to the loss caused by the cross-coupling effect triggered by backscattering. To examine the detailed process of compensating for the Sagnac effect in the RLG, the equation for the interference of two beams with a phase difference of ψ is expressed as

(14) $$E=E_{0}e^{i\left(\omega t+\phi_{0}\right)}+E_{0}e^{i\left(\omega t+\phi_{0}+\psi \right)}=E_{0}e^{i\left(\omega t+\phi_{0}\right)}F,\;$$

where $E_{0}$ is the amplitude, ω is the angular frequency; $\phi_{0}$ is the initial phase of the light wave; and $F=1+e^{i\psi }$ is the normalized interference. The intensity-type interference signal is obtained by $FF^{*}$, where $F^{*}$ is the complex conjugate of F. The frequency difference $\dot{\psi }/2\pi$ was extracted from the measured interference signal. A complex error function ϵ is defined as

(15) $$\epsilon \equiv e^{i\psi }-e^{i\psi_{i}}=Re^{i\Phi }.\;$$

Then, the normalized interference can be rewritten as

(16) $$F=1+e^{i\psi }=1+e^{i\psi_{i}}+Re^{i\Phi }.\;$$

Therefore, Equation (16) can be interpreted as two complex functions formed through interference taking the form of causing interference again at a higher level. Broadly stated, when the normalized interference F is measured using a photodetector, frequency components corresponding to the difference between frequency components due to $e^{i\psi_{i}}$ and frequency components appearing due to $\epsilon =Re^{i\Phi }$ are provided as an output. In conclusion, ϵ has a role as a compensator of the Sagnac effect, and the corresponding compensation is realized through the interference at a higher level.

## 5. Analysis of Sagnac Effect Compensations with the Error Function

In this section, we summarized the results of the analysis of the Sagnac effect compensation related to the frequency lock-in characteristic using the error function **ϵ** defined in Section 4.

First, to determine the frequency component corresponding to ϵ, we defined $R_{a}$ by taking the average in the interval during the dithering period $T_{D}=2\pi /\omega_{D}$ for the alternating component of R, as expressed in Equation (17).

(17) $$R_{a}\equiv \langle R-R_{ave}\rangle {}_{T_{D}}\;,$$

where $R_{ave}$ is the overall average of R over the observation time. In the case of Φ, it did not have meaningful information to compensate for the Sagnac effect owing to the high-frequency component caused by dithering. Therefore, to compensate for the Sagnac effect, the time-varying property of ϵ approximately followed $R_{a}$.

**Figure 2:**
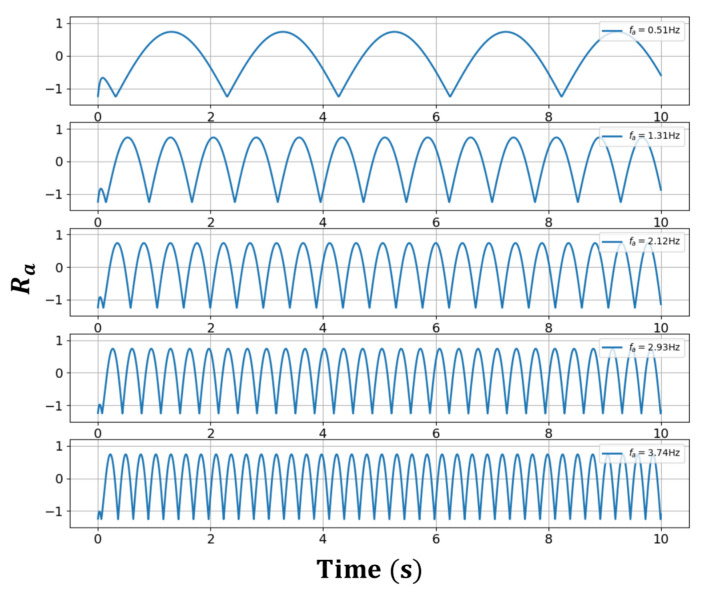
$R_{a}$ within the lock-in area.

**Figure 3:**
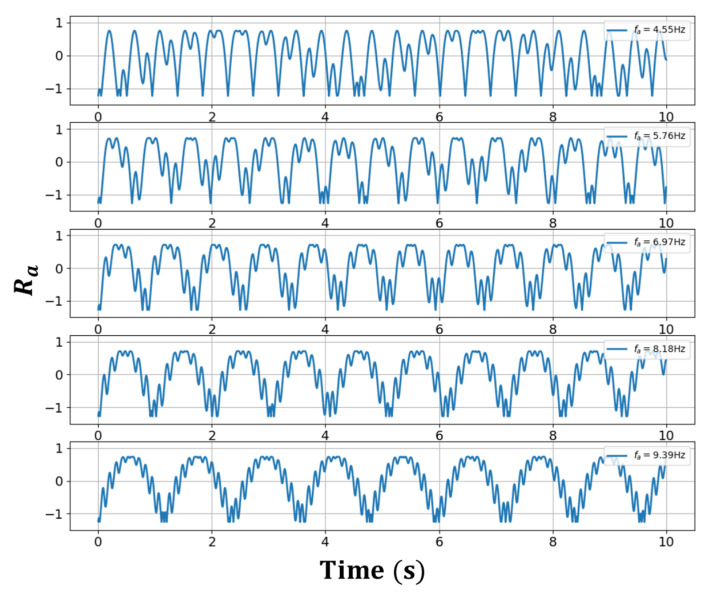
$R_{a}$ outside the lock-in area.

Figure 2 and Figure 3 show the $R_{a}$ oscillating characteristics with respect to time as a changes within and outside the frequency lock-in region, respectively. To obtain $R_{a}$**,** for ψ- and $\psi_{i}$- related data, the same conditions as in Figure 1 were used. The frequency component that dominates $R_{a}$ increased in proportion to the increases in a in the frequency lock-in region, whereas it was generally lowered in inverse proportion to the increase in a when it was outside the frequency lock-in region. When recalling the typical frequency lock-in characteristic that the RLG has a zero-output value when a is within the frequency lock-in region, whereas the outputs of the RLG approach a as a result of increases in a when it is outside the frequency lock region, it was inferred that the frequency component that dominates $R_{a}$ acted in a form that compensates for the given Sagnac effect. It is particularly noteworthy from Figure 2 and Figure 3 that, outside the frequency lock-in region, different frequency components are mixed, unlike inside the frequency lock-in region. It was observed that phase transitions occurred based on the frequency lock-in threshold, and the mechanism for compensating the Sagnac effect outside the frequency lock-in region was not simple, unlike inside the frequency lock-in region where the Sagnac effect was completely compensated by a single frequency component.

**Figure 4:**
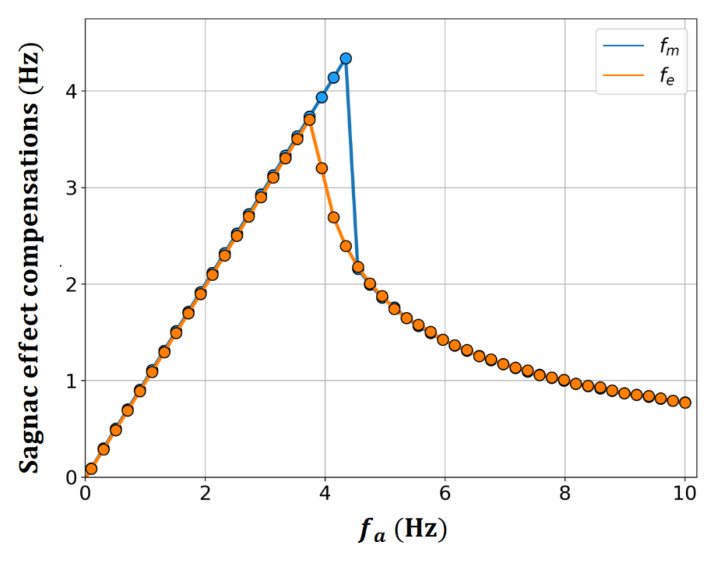
Sagnac effect compensations with $f_{m}$ and $f_{e}$ as a function of $f_{a}=a/2\pi$.

Figure 4 shows the frequency $f_{m}$ at the point with the maximum value when $R_{a}$ is analyzed in the frequency domain using a Fast Fourier Transform (FFT), and the difference in the output of the RLG $f_{e}$ for the case where b is nonzero compared to the ideal case of Figure 1. From Figure 4, it is observed that $f_{m}$ and $f_{e}$ are in good agreement overall, but there is a significant difference near the frequency lock-in threshold.

**Figure 5:**
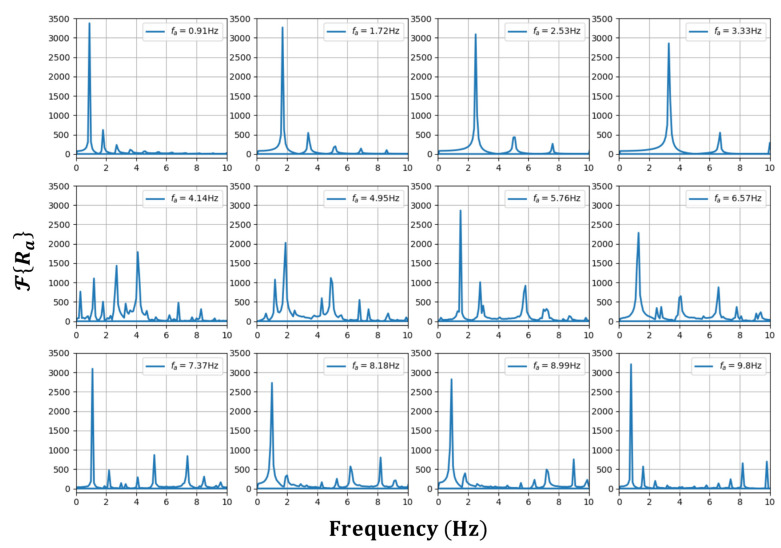
Analyzed frequency components of $R_{a}$ with changes in $f_{a}=a/2\pi$.

To improve the understanding of this phenomenon, Figure 5 shows the FFT analysis results for $R_{a}$, $ℱ\left\{R_{a}\right\}$ according to the change in a. When a is away from the frequency lock-in threshold, $f_{m}$ moves in a linearly increasing fashion with respect to changes in a when a is within the frequency lock-in region, and nonlinearly decreases when ***a*** is outside the frequency lock-in region, so that $f_{m}$ is equivalent to $f_{a}$ and $f_{e}$. However, at $f_{a}$ = 4.14 Hz, which is near the frequency lock-in threshold, $f_{m}$ still shifts linearly with the change in a and coincides with $f_{a}$, while $f_{e}$ in Figure 4 has a lower value of ~2.69 Hz. This difference is due to the combined action of the following two characteristics.

First, as a exceeded the frequency lock-in threshold, the harmonic components were generated. Although this phenomenon disappeared as a increased, it became more pronounced as a approached the frequency lock-in threshold. The second feature is the intrinsic method, in which the RLG recognizes the frequency difference owing to the Sagnac effect. As mentioned in Section 3, the RLG output expressed by Equation (13) represents the average frequency, and, for this reason, $f_{e}$ also corresponds to the average frequency corresponding to the center of gravity of the related frequency distribution, not a single frequency. Therefore, when $R_{a}$ was dominated by one specific frequency component, there was no significant difference between $f_{m}$ and $f_{e}$. However, when various frequency components were evenly and widely distributed as in $f_{a}$ = 4.14 Hz, there was a significant difference between $f_{m}$ and $f_{e}$.

It is evident from the detailed graphs in Figure 5 that a frequency component that increases linearly with an increase in $f_{a}$ loses its qualification of $f_{m}$ at some point because its magnitude decreases as it crosses the frequency lock-in threshold. This contributed to the Sagnac effect compensation through $f_{e}$. The correlation between the frequency characteristics that increased linearly with the increase in $f_{a}$ and the frequency characteristics that increased nonlinearly with the decrease in $f_{a}$ together with the generation of multiple frequency components occurring near the frequency lock-in threshold, was considered a key factor in determining the frequency lock-in dynamics.

## 6. Discussion and Conclusions

The harmonic characteristics that occurred as phase transitions near the critical point were also confirmed in recent studies [17]. The results of this study showed that the RLG frequency lock-in phenomenon is connected to the optics and photonics research field that is receiving considerable attention, which is called an exceptional point in the physical situation with a non-Hermitian Hamiltonian (NHH) [18,19,20,21,22]. The methodology regarding the Sagnac effect compensation for the frequency lock-in phenomenon presented in this study has many similarities with the research field of the exception point. For example, one of the basic concepts describing the field is related to the phase transition observed in a structure in which the properties of gain and loss are combined. In this case, the Sagnac effect corresponds to the gain, the Sagnac effect compensation related to the frequency lock-in phenomenon corresponds to the loss, and the nonlinear characteristics occurring near the frequency lock-in threshold correspond to the phase transition. This field is related to finding a new direction to improve the performance of RLG, and the study of realizing a precise sensor using the sensitivity enhancement phenomenon that appears at the exception point is a promising future-oriented research direction related to the RLG [23,24].

From another perspective, it is noteworthy that the results in this work are basically unlimited to the conventional gyroscopes based on He-Ne gas lasers. This is because the integrative analysis with the complex error function gives a chance to treat critical points about the lock-in phenomenon in the fundamental and principal aspects; the results have sufficient room for help in the other laser gyroscope technologies based on such as semiconductor ring resonators that also usually suffer from the lock-in and mode competition effects [25]. In addition, the nonlinear characteristics that occur when encircling the critical point analogous to the dithering situation with the RLG are not only meaningful when considering fundamental research in the field of physics but also have high research value because of their application potential in various fields [26,27]. Therefore, future research on the RLG in connection with the relevant field is meaningful in that it can pioneer and develop a new research field with locked phases.

In summary, the main sources and mechanisms of the locked states of the RLG with dithering were thoroughly reviewed and analyzed through numerical experiments. The analyzed results showed typical phase-transition characteristics based on the frequency lock-in threshold. The analyzed results showed that the essential physical properties are strongly related to Sagnac effect compensation. To fundamentally understand the frequency lock-in phenomenon more deeply, it was concluded that additional integrative and generalized research on peculiar phenomena appearing near the frequency lock-in threshold is necessary.

## Data Availability

Data are available upon reasonable request to the corresponding author.
